# Comparative Analysis of Dopaminergic and Cholinergic Mechanisms of Sensory and Sensorimotor Gating in Healthy Individuals and in Patients With Schizophrenia

**DOI:** 10.3389/fnbeh.2022.887312

**Published:** 2022-06-30

**Authors:** Andrey T. Proshin

**Affiliations:** P. K. Anokhin Institute of Normal Physiology, Moscow, Russia

**Keywords:** dopaminergic and cholinergic mechanisms, prepulse inhibition, sensorimotor gating, sensory gating, schizophrenia

## Abstract

Sensory and sensorimotor gating provide the early processing of information under conditions of rapid presentation of multiple stimuli. Gating deficiency is observed in various psychopathologies, in particular, in schizophrenia. However, there is also a significant proportion of people in the general population with low filtration rates who do not show any noticeable cognitive decline. The review article presents a comparative analysis of existing data on the peculiarities of cholinergic and dopaminergic mechanisms associated with lowering gating in healthy individuals and in patients with schizophrenia. The differences in gating mechanisms in cohorts of healthy individuals and those with schizophrenia are discussed.

## Introduction

The mechanisms of sensory and sensorimotor gating provide adaptive patterns of responses to multiple stimuli presented with rapid succession (Swerdlow et al., 2000) working at the stages of pre-attention and early attention. A lot of studies reported the impairment of these gating mechanisms in patients with schizophrenia (Adler et al., 1982, 2004; Baker et al., 1987; Light and Braff, 1999; Braff et al., 2001; Perry et al., 2001; Cadenhead and Braff, 2002; Swerdlow et al., 2008; San-Martin et al., 2020).

In translational studies, the model of the suppression of P50 auditory event-related potential (AEP) in a condition–test paradigm is considered the putative measure of sensory gating. A deficit of sensory gating may result in sensory overflood and inappropriate assessment of stimuli salience (Evans et al., 2007).

To quantify sensorimotor gating, a model of prepulse inhibition (PPI) of the startle response is applied (Graham, 1975; Braff et al., 2001; Swerdlow et al., 2008). In this experimental paradigm, a quantitative measure of sensorimotor gating is a reduction in the startle reaction magnitude due to weak sensory pre-stimulation delivered with a short (<500 ms) stimulus onset asynchrony (SOA). PPI is regarded as a complex measure reflecting the early mechanisms of stimuli processing and action selection (Blumenthal, 2014).

The individual characteristics of sensory and sensorimotor gating are assumed to contribute to higher cognitive functions (working memory, voluntary attention and executive functions), which are deficient under psychopathological conditions (Bitsios and Giakoumaki, 2005; Holstein et al., 2011; Toyomaki et al., 2015). P50 suppression and PPI are impaired in patients with schizophrenia (Braff et al., 2001; Swerdlow et al., 2008; Braff, 2010; San-Martin et al., 2020, 2022). The measures display a significant degree of heritability (Clementz et al., 1998; Cadenhead et al., 2005; Hasenkamp et al., 2010; Earls et al., 2016; Togay et al., 2020; Li et al., 2021) and are considered to be sendo phenotypes of schizophrenia (Turetsky et al., 2007; DiLalla et al., 2017).

At the same time, numerical studies found the impairment of sensory and/or sensorimotor gating in some other mental and neurological disorders, such as Huntington's disease (Swerdlow et al., 1995), Tourette's syndrome (Swerdlow, 2013; Buse et al., 2016), obsessive–compulsive disorder (Ahmari et al., 2012), bipolar disorder (Baker et al., 1990; Sánchez-Morla et al., 2016; Mao et al., 2019; Atagun et al., 2020), and autism (Crasta et al., 2021). The deficit of PPI and P50 suppression was found in the prodromal stages of Alzheimer's disease and can be used to differentiate the early stages of this pathology from mild cognitive impairment (Jafari et al., 2020).

These data raise questions about the existence of mechanisms of gating impairment that are common or specific for different psychopathologies. Moreover, a lot of clinically unaffected individuals without substantial cognitive deficit display a low level of sensory or sensorimotor gating (Hasenkamp et al., 2010; Knott et al., 2013). Neuroimaging studies showed that patterns of associations between gating rates and the activity of different brain structures are distinct in healthy individuals and in patients with schizophrenia (Kumari et al., 1996; Williams et al., 2011; Naysmith et al., 2021).

These data suggest specificity in the contribution of different neurotransmitter system activities to deficiency of information gating in healthy subjects and in various psychopathologies. Among others, the study of this problem is important for the development of potential therapeutic approaches (Swerdlow et al., 2000). One of the first steps in this direction should be a comparative analysis of the neurotransmitter mechanisms of gating processes in healthy subjects and patients with schizophrenia.

Two main hypotheses about neurotransmitter disorders in schizophrenia consider the abnormal functioning of dopaminergic and glutamatergic signaling. The interaction of glutamatergic and dopaminergic systems in the prefrontal cortex plays a significant role in the mechanisms of attention and executive control (Aquila and Citrome, 2015; Storozheva et al., 2015). Of note, the results of the animal studies showed relatively independent pathways for the participation of glutamatergic and dopaminergic transmission in the mechanisms of PPI (Keith et al., 1991; Callahan et al., 2014; Oliveras et al., 2017). At the same time, the close interaction of dopaminergic and cholinergic activities on sensory and sensorimotor gating has been documented in several studies (De la Salle et al., 2013). It should be noted that only five associations of genetic variants with sensory or sensorimotor gating were confirmed in repeated studies of independent samples. Three of these five polymorphisms are located in genes that regulate dopaminergic (COMT) and cholinergic (CHRNA3 and CHRNA7 acetylcholine receptors) neurotransmission (Rovn et al., 2020).

This work aimed to provide a comparative analysis of the existing data on the involvement of dopaminergic and cholinergic signaling systems in sensory (P50 suppression) and sensorimotor (PPI) gating in populations of mentally healthy people and patients with schizophrenia. The most attention was paid to the results of genetic and pharmacological studies.

## Prepulse Inhibition of the Startle Reaction

### General Definition and Relevant Neural Circuits

The startle reaction is a generalized defensive reaction evoked by a sudden intensive stimulus. In humans, startle is mainly estimated by the blink component. The most often used model is acoustic startle reaction (ASR) evoked by sudden sounds with intensity >95 dB. Prepulse inhibition (PPI) is the phenomenon of suppression of the amplitude of response to the intensive (main) stimulus by weaker (<90 dB) sounds (prepulse) presented with a short interval before the main signal. In different studies, the intervals between prepulse and the main stimulus (stimulus onset asynchrony—SOA) varied within the range from 10 to 300 ms (Braff et al., 2001; Swerdlow et al., 2008, 2018).

PPI is observed across species, including invertebrates (Engel and Hoy, 1999; Frost et al., 2003), fishes (Burgess and Granato, 2007), and mammals (Swerdlow et al., 2008). In mammals, the primary neural circuit that mediates the acoustic startle response originates from cochlear root neurons, which send projections to the caudal pontine reticular nucleus (PnC). Axons of PnC provide the excitation of motor neurons to produce the startle response (Lingenhöhl and Friauf, 1994). The PnC also receives modulatory projections from pedunculopontine and laterodorsal tegmental nuclei, which also receive acoustic information from the inferior and superior colliculi (Fendt et al., 2001). A lot of structures participate in the modulation of ASR, such as the striatum and pallidum, ventral tegmental area, hippocampus, amygdala, raphe nuclei, thalamus, and prefrontal cortex. It can be suggested that some of multiple circuits involved in startle modification differentially participate in PPI at various SOAs (Gómez-Nieto et al., 2020).

The association between PPI at SOA 120 ms and activity in the striatum, thalamus, insula, hippocampal, temporal, inferior frontal and inferior parietal regions was found in fMRI studies in healthy controls and in patients with schizophrenia (Kumari et al., 2007).

The activity of dopaminergic and cholinergic synapses largely determines the functions of both the main PPI circuits and the structures that participate in the modulation of PPI (Davis, 1980; Chandler et al., 2013; Prager and Plotkin, 2019).

### PPI and Brain Dopaminergic Neurotransmission

#### Genetic Research

Although changes in the activity of the dopaminergic system are considered as one of the main mechanisms of the aetiopathogenesis of schizophrenia, only a small number of polymorphisms in genes that regulate dopaminergic neurotransmission have been found to exert significant effects on sensorimotor gating (Montag et al., 2007; Roussos et al., 2008a,b; Quednow et al., 2018; Rovn et al., 2020). Only one of those, namely polymorphism rs4680 in the catechol-O-methyl transferase (COMT) gene, has been studied both in healthy controls and in patients with schizophrenia.

COMT is the main enzyme that degrades dopamine in the prefrontal cortex (Chen et al., 2004). Polymorphism rs4680 (Val158Met substitution) is associated with the enzymatic activity of COMT, meaning that the presence of the Val allele leads to an increased dopamine degradation rate and a decrease in its level in the prefrontal cortex. The effect of the COMT rs4680 polymorphism on PPI in healthy volunteers has been shown in a number of studies. The association of rs4680 with PPI in the general population was confirmed by a previous meta-analysis (Quednow et al., 2018). In the sample of Greek/Caucasian men, the highest level of PPI was observed in carriers of the methionine homozygote and the lowest was in carriers of the Val/Val genotype, while carriers of the Val/Met variant displayed intermediate values. Polynomial contrasts showed a linear relationship between PPI levels and Val allele load (Roussos et al., 2008b). Similar results were obtained in a Russian population of healthy men (Kirenskaya et al., 2015). In the study of Quednow et al. (2009), the effect of rs4680 on PPI was only significant in the male cohort, but not in the total gender-mixed Caucasian sample of healthy volunteers. In all of these studies of rs4680, the effects of rs4680 on PPI were found at SOAs 60 and 120 ms.

The study in young-adult Chinese women revealed the positive association of PPI (at SOA 120 ms) with the Met allele and also showed the stabilizing effect of the Met allele under conditions of menstrual-cycle-dependent PPI variation (Wu et al., 2019).

Montag et al. (2007) did not show the effect of rs4680 on PPI at SOA 120 ms in the cohort of mentally healthy Caucasian females.

In summary, the existing literature data allow an association to be proposed between the valine allele of the rs4680 polymorphism with a low level of PPI in a population of mentally healthy males. The question of the existence of such an association in mentally healthy females can be considered open.

Data on the effects of the COMT polymorphism rs4680 on PPI obtained in cohorts of patients with schizophrenia are contradictory. In a gender-mixed Chinese sample of patients with first-episode schizophrenia, no effect of rs4680 on PPI at SOA 120 ms was found (Liu et al., 2013). The study in Russian male patients with schizophrenia also failed to reveal any effects of rs4680 on PPI tested at SOAs 60 and 120 ms (Quednow et al., 2010; Kirenskaya et al., 2015). Quednow et al. (2009) found the effect of rs4680 on PPI at SOA 120 ms in a gender-mixed Caucasian cohort of patients with schizophrenia. In this study, the analyses of polynomial contrasts across the three genotype groups revealed a significant quadratic trend, unlike healthy controls who displayed a linear trend (see above). Accordingly, carriers of the Met/Met genotype displayed higher PPI levels compared to carriers of the Val/Met genotype, while the difference between Met/Met and Val/Val and between Val/Met and Val/Val was not significant. In summary, analysis of the available data suggests that the effects of the rs4680 polymorphism on the level of PPI are significantly less pronounced in patients with schizophrenia than in healthy subjects.

#### Pharmacological Studies

Most of the initial data on the effects of dopamine agonists and antagonists on PPI have been obtained in animal experiments. As the data were accumulated, comparative analysis showed that the effects of dopamine agonists and antagonists on PPI can differ in animals and humans. Accordingly, studies in patients and healthy controls are important for verification of the translational models of schizophrenia spectrum and other mental disorders.

In healthy individuals, the mixed D1/D2 agonist apomorphine decreased PPI in high-intensity (85 dB) prepulse trials and enhanced sensorimotor gating in persons with low baseline PPI under conditions of low (75 dB) prepulse intensity (Schellekens et al., 2010). In this study, the effects of apomorphine on the stability and selectivity of attention were estimated in the continuous performance test (AXCPT version). In brief, subjects were instructed to detect targets and non-targets within a stream of presented letters. Targets were the letter X when they followed the letter A and X following non-A cues were non-targets. The response to the non-target letter A is usually regarded as a commission error. In turn, the number of commission errors is a negative measure of the selectivity of attention. The authors found the impairment of AXCPT performance after apomorphine administration and an association between the high selectivity of attention (low commission errors) with *reduced* baseline sensorimotor gating at SOA 100 ms and low prepulse intensity. No studies have been conducted on the effects of apomorphine on PPI in patients with schizophrenia.

Data on the effects of D2 agonists on PPI in healthy persons are contradictory. Hutchison and Swift (1999) found a reduction in PPI at an SOA of 120 ms after the acute administration of 20 mg dexamphetamine. In another study with the same dose of amphetamine (Swerdlow et al., 2003), a significant decrease in PPI was observed at an SOA of 10 and 20 ms, but not at 30 or 60 ms; the decrease in PPI at an SOA of 120 ms was at the level of trend.

Kumari et al. (1998) found that the acute administration of d-amphetamine decreased PPI across 30-ms−60-ms−120-ms intervals in smoking individuals but not in the total sample of healthy males. However, the sample in this study was not large enough to estimate drug x SOA interaction.

A number of studies in healthy individuals did not find any substantial effect of amphetamines on PPI (Swerdlow et al., 2002a; Talledo et al., 2009; Chitty et al., 2014).

Swerdlow et al. (2002b) found that another D2 agonist bromocriptine decreased PPI at SOA 120 ms (but not at SOA 60 ms) and increased PPI at SOA 20 ms. The disruption of PPI at SOA 120 ms after bromocriptine administration was found also by Abduljawad et al. (1998, 1999) and Martin-Iverson (1999).

In the study of Swerdlow et al. (2002b), the D2 agonist amantadine decreased PPI at an SOA of 60 ms and 120 ms, but increased PPI at an SOA of 10 ms. In another study by these authors, the decrease in PPI at SOA 100 ms was found 15 min after amantadine administration; however, in the course of successive test sessions (from first to fifth with 15-min intervals), PPI increased to a level above that of the control group. Whether this trend is the result of a change in the effect of the drug over time or is related to the effect of amantadine on the effectiveness of retraining needs to be investigated.

When interpreting the effect of amantadine, it should be taken into account that it is also an NMDA glutamate receptor antagonist (Raupp-Barcaro et al., 2021).

In patients with schizophrenia, acute amphetamine enhanced PPI at an SOA of 60 ms (but not at an SOA of 120 ms) and increased it to a level comparable to that of control values. The increase in PPI after amphetamine administration was more pronounced in carriers of the methionine allele of rs4680 and displayed a correlation with positive symptoms (Swerdlow et al., 2018).

In healthy individuals, some authors (Abduljawad et al., 1998; Martin-Iverson, 1999; Oranje et al., 2004a) found the disruption of PPI estimated at an SOA of 120 ms after the administration of the D2 antagonist haloperidol. At the same time, haloperidol attenuated the disruptive effect of bromocriptine on PPI (Abduljawad et al., 1999; Martin-Iverson, 1999). Csomor et al. (2008) found that haloperidol had no effect on PPI in subjects with low baseline levels of PPI but attenuated PPI in subjects with high baseline gating levels. In this study, a positive association of high baseline PPI with the successful performance of the spatial working memory task was observed. Kumari et al. (1998) reported that haloperidol disrupted PPI in normal smoking subjects but had no effect on non-smoking individuals; however, the modest sample size of smoking participants in this work made it impossible to estimate the significance of the treatment x SOA interaction on PPI. Some studies reported no effect of haloperidol on PPI in healthy volunteers (Abduljawad et al., 1999; Graham et al., 2004).

None of the studies revealed the effect of haloperidol on PPI at different SOAs in patients with schizophrenia (Duncan et al., 2003; Kumari et al., 2007; Wynn et al., 2007). Also, long-term (2–6 weeks) treatment with amisulpride showed no effects on PPI, either in the cohort of initially antipsychotic-naive, first-episode schizophrenia patients or in mentally healthy individuals (Düring et al., 2014).

Thus, the data of both genetic and pharmacological studies suggest differences in dopaminergic mechanisms that determine the effectiveness of sensorimotor gating in healthy subjects and in patients with schizophrenia.

### PPI and Brain Cholinergic Activity

#### Genetic Studies

Studies of the association between polymorphic variants of the genes involved in cholinergic neurotransmission and the individual levels of sensorimotor gating revealed significant effects for the polymorphisms rs1051730 and rs1317286 in the gene encoding the alpha 3 subunit of nicotinic receptors (CHRNA3) (Petrovsky et al., 2010). In the human brain, high densities of CHRNA3 are observed in the frontal, temporal, and parietal cortex, thalamus, striatum, and hippocampus (Gotti and Clementi, 2004), that is in the structures that are considered to be involved in the mechanisms of PPI.

In healthy subjects, the effects of the rs1051730 and rs1317286 polymorphisms of the CHRNA3 gene on PPI were described by a linear trend (CC>CT>TT and AA>GA>GG, accordingly), while a significant quadratic trend was observed in patients with schizophrenia with regard to the maximum values of PPI in CT/GA heterozygotes (Petrovsky et al., 2010).

In healthy individuals, the effects of rs1051730 and rs1317286 on PPI were assessed at SOAs of 30 ms, 60 ms, and 120 ms; however, the authors did not report any significant interaction of interval x genotype effects. In patients with schizophrenia, the effects of the rs1051730/rs1317286 haplotype were estimated at SOA 120ms (Petrovsky et al., 2010).

In another study by the same authors, the effect of acute nicotine administration on PPI was evaluated in mentally healthy carriers of different variants to rs1051730. Nicotine has been shown to increase PPI in carriers of the TT variant (associated with low baseline PPI) and decrease it in carriers of the C allele. These effects of nicotine were observed at SOAs of 120 and 240 ms but not at an SOA of 30 or 60 ms (Petrovsky et al., 2010).

Nicotinic receptors that contain alpha 4 (CHRNA4) subunit display high affinity for nicotine and are expressed in the cortex, hippocampus, basal ganglia, and cerebellum (Gotti and Clementi, 2004). In the striatum, CHRNA4 is found in the dopaminergic neurons and is involved in the interaction of dopamine and cholinergic mechanisms of attention and working memory (Cools et al., 2007; Markett et al., 2010; Giessing et al., 2011). Also, it was found that the pattern of thalamo-cortical connectivity is associated with polymorphism of the CHRNA4 gene (Winterer et al., 2007; Giessing et al., 2011).

In the study of Shi et al. (2016), the effects of three polymorphisms in the CHRNA4 gene: rs3746372, rs1044396 and rs3787140 on PPI at an SOA of 30ms, 60ms, and 120ms, were estimated in Chinese samples of healthy volunteers and patients with schizophrenia.

In healthy individuals, no significant effects of the studied polymorphisms on PPI were found. In patients with schizophrenia, the level of PPI at an SOA of 120 ms was significantly lower in patients with the GG genotype of rs3746372 and the TT genotype of rs1044396 compared to other variants (Shi et al., 2016). Thus, the specific participation of CHRNA4 in mechanisms of PPI in patients with schizophrenia could be proposed.

In contrast to CHRNA3 and CHRNA4, no effects of the polymorphism of CHRNA7 on PPI were found in healthy individuals or in patients with schizophrenia (Liu et al., 2013; Bertelsen et al., 2015).

#### Pharmacological Studies

The data obtained in a number of studies indicate that the acute administration of nicotine, as well as its chronic consumption (smoking), increases the PPI in both healthy individuals and in patients with schizophrenia (Kumari et al., 1996, 1997; Postma et al., 2006; Baschnagel and Hawk, 2008; Rabin et al., 2009; Song et al., 2014), although some studies reported a decrease in the PPI after smoking high nicotine cigarettes compared to PPI after smoking low nicotine ones (Hutchison et al., 2000). George et al. (2006) found that the acute withdrawal from smoking impairs PPI in patients with schizophrenia but not in healthy controls. In addition, smoking reinstatement reverses the deficit of PPI caused by abstinence in patients without any effect on sensorimotor gating in control smokers. Mecamylamine, a preferential antagonist of subtype α(4)β(2) and α(3)-containing cholinergic receptors, prevented the effect of smoking on PPI in patients with schizophrenia without any effect on the control group. In the study by Duncan et al. (2001), a stimulating effect of nicotine on PPI after nocturnal withdrawal was found in healthy smoking subjects. However, taking into account the design of the experiment, it is also likely that the authors observed a direct effect of acute nicotine on PPI, regardless of abstinence.

Thus, it can be assumed that there are differences in the effects of the withdrawal and recovery of nicotine consumption on PPIs in healthy individuals and patients with schizophrenia; this difference is dependent on the activity of nicotine receptors.

The results obtained in the study of Postma et al. (2006) indicate multiple interactions of diagnosis, nicotine, SOA and time elapsed after nicotine administration on PPI.

Rabin et al. (2009) found that PPI is positively associated with cognitive performance (Wisconsin Card Sorting Test) in smoking patients with schizophrenia but not in non-smoking patients or healthy individuals.

In summary, although the existing data are not numerous, they indicate the difference in the genetic mechanisms that determine the effectiveness of sensorimotor gating in healthy subjects and in patients with schizophrenia. In particular, the difference in the rules of allele interactions between patients with schizophrenia and controls was revealed when the effects exerted on PPI by polymorphism rs4680 in the COMT gene, as well as polymorphisms rs1051730 and rs1317286 in the CHRNA3 gene, were estimated. Also, the effects of polymorphisms rs3746372 and rs1044396 in the CHRNA4 gene were only observed in schizophrenia, but not in controls. The results of pharmacological studies show that acute withdrawal from smoking and the reinstatement of nicotine consumption exert an effect on PPI only in patients with schizophrenia, but not in healthy people. Moreover, these effects that are specific to patients are mediated by α(4)β(2) and α(3)-containing cholinergic receptors, which is in agreement with the results of previous genetic studies.

The available results of pharmacological studies show that the dopaminergic mechanisms of sensorimotor gating in humans are almost as mysterious as the dopaminergic mechanisms of schizophrenia.

The effects of dopamine receptor agonists, such as apomorphine, bromocriptine, and amantadine, have not been studied in patients with schizophrenia, which makes comparative analysis impossible. However, data obtained in a population of mentally healthy individuals deserve analysis. Of great interest is the pronounced dependence of the effects of these drugs on SOA length. Surprisingly, both agonists (amphetamine, bromocriptine and amantadine) and antagonists (haloperidol) of D2 receptors reduce PPI in healthy subjects. The most frequent decrease in PPI after D2 agonist administration was observed at an SOA of 120 ms.

The effects of D2 agonists on PPI at SOAs <30 ms displayed an association with their effects on dopamine synthesis (detected *in vitro*). Thus, amphetamine, which elevates dopamine synthesis in presynaptic endings, significantly decreased PPI at an SOAs of 10 ms and 20 ms, while bromocriptine, which suppresses the synthesis of dopamine in synaptosomes, increased PPI at an SOA of 20 ms (Tissari, 1988; Tissari and Lillgäls, 1993; Swerdlow et al., 2003). These data suggest the mechanisms of “early” PPI may depend on the activity of dopaminergic projections to pontine and medullary structures (Kitahama et al., 2000; Sharma et al., 2018).

Importantly, there were no effects of amphetamine on “early” PPI in patients with schizophrenia (Swerdlow et al., 2018).

It should be noted that PPI at an SOA of 120 ms displayed the highest level of heritability (Roussos et al., 2016). At the same time, data from studies evaluating the differences in PPI between patients and healthy individuals with various SOAs are inconsistent.

Thus, most authors reported that PPI deficits in schizophrenia were observed at an SOA of 60-ms intervals but not at 30 or 120 ms (Wang et al., 2013; Swerdlow et al., 2015, 2018). Some other studies revealed the lowering of PPI at SOA 120 ms in patients with schizophrenia (Hong et al., 2007; Wynn et al., 2007). Ludewig and Vollenweider (2002) found that patients with schizophrenia with deficit syndrome showed an impairment of PPI at an SOA of 60 ms, while patients with a non-deficit syndrome showed a PPI that was lowered at longer SOAs (up to 240 ms). In this regard, of particular interest are the data obtained by Swerdlow et al. (2017), who found that amphetamine caused an increase in PPI at an SOA of 60 ms in patients with schizophrenia and the level of enhanced PPI positively correlated with positive symptoms. Positive correlations between PPI level and hallucinations were also found in schizophrenic patients (Storozheva et al., 2015). At that, Cadenhead (2011) assessed PPI in medication naive early psychosis are at risk psychosis subjects. She found that subjects who later developed psychosis had a greater PPI at an SOA of 60 ms compared to the healthy controls.

Thus, dopaminergic mechanisms can be involved in compensatory processes in conditions of psychosis development. The association of the effects of amphetamine on PPI with the polymorphism of COMT, discovered by Swerdlow et al. (2018), suggests the dependence of such compensatory processes on activity of the prefrontal cortex. It is also worth noting that the modulating effect of amphetamine on PPI is only observed in animals with a mature prefrontal cortex (Mena et al., 2021). Such cortical-dependent compensatory processes may be of particular interest in understanding the pathogenesis of schizophrenia.

It is believed that sensorimotor gating protects contextual information from the interfering effects of extreme signals, providing working memory processes. However, excessive attention to contextual information (extremely high PPI) can also disturb mental functions and behavioral processes. Indeed, while Bitsios et al. (2006) found positive associations of PPI with successful strategy formation and the speed of spatial memory task performance, Schellekens et al. (2010) revealed an association between high selectivity of attention in continuous performance tasks and reduced levels of gating.

These data raise questions surrounding the existence of optimal level of gating, and comprehensive comparison of healthy individuals and patients with schizophrenia with high and low PPI levels should be provided. Such a systematic study may include the analysis of relevant genetic polymorphisms, as well as psychophysiological and cognitive tests.

The assumption of the participation of the dopaminergic system in maintenance of the optimal level of sensorimotor gating is also supported by the observed effects of haloperidol, which increase the PPI in healthy individuals with initially low levels and reduce in healthy individuals with initially high PPI levels.

## Auditory-Evoked Potential P50

### General Definition and Relation to the Activity of Nerve Circuits

Auditory sensory gating is usually assessed in a paired-click paradigm that involves the presentation of two identical clicks (S1 and S2). Estimation of the auditory event-related potential at around 50 ms post-click (mid-latency P50 component) reveals a reduced amplitude in response to S2 (testing stimulus) relative to the amplitude in response to S1 (conditioning stimulus). The optimal interval between S1 and S2 is 500 ms.

### Dopaminergic Mechanisms of Sensory Gating

#### Genetic Studies

Neuronal correlates of sensory gating were found in the hippocampus, temporo-parietal, and prefrontal cortex (Grunwald et al., 2003; Freedman et al., 2020). The mechanisms of gating *per se* are mainly associated with hippocampal and prefrontal activity (Yvert et al., 2001; Korzyukov et al., 2007; Freedman et al., 2020). Accordingly, polymorphism rs4680 in the COMT gene was proposed to be associated with the efficacy of frontal gating mechanisms.

Some studies revealed the effect of rs4680 genotype on sensory gating in schizophrenia patients with significantly lower P50 suppression rate in Val/Val carriers (Lu et al., 2007; Liu et al., 2013). The study of Lu et al. (2007) included the samples of patients and healthy individuals; the latter did not display any significant effect of rs4680 genotype on sensory gating.

The association of the Val/Val genotype with decreased P50 gating was found in healthy individuals by De la Salle et al. (2013).

The opposite result was obtained by Storozheva et al. (2019) who found the maximum amplitude for the first stimulus in the pair (A1) and the highest level of P50 suppression in healthy carriers of Val/Val genotype. No effects of COMT on P50 were found in patients with schizophrenia.

Some authors failed to find any substantial effects of the rs4680 polymorphism in the COMT gene on P50 suppression rate in patients with schizophrenia or in healthy persons (Shaikh et al., 2011; Demily et al., 2016; Mao et al., 2016).

Therefore, it is not possible to draw an unambiguous conclusion about the similarity or difference between the effects of rs4680 on sensory gating in patients and healthy people.

It is worth noting that a significant effect of the COMT genotype was observed in healthy persons for another measure of sensory gating—N100 suppression, with the poorer gating in Met/Met individuals compared to carriers of the Val allele (Majic et al., 2011).

Millar et al. (2011) and Knott et al. (2010) found the effects of polymorphisms in the dopamine transporter (VNTR) and D2 receptor (TaqIA) genes on P50 gating in healthy individuals, but similar studies have not been conducted in patients with schizophrenia.

#### Pharmacological Studies

Data on the effects of compounds that directly affect dopaminergic neurotransmission on sensory gating are controversial. Light et al. (1999) found that amphetamine disrupted P50 suppression in healthy persons. In a study by Csomor et al. (2008), it was found that haloperidol increased P50 suppression in healthy subjects exhibiting low P50 gating and disrupted P50 suppression in individuals with initially high P50 gating rates. In another study, the disruptive effect of haloperidol in healthy persons was observed (Witten et al., 2016). A lot of studies have found no effects on PPI of substances that affect dopaminergic transmission (Arango et al., 2003; Oranje et al., 2004b; Düring et al., 2014). Overall, the dopaminergic mechanisms of sensory gating remain poorly understood.

### Brain Cholinergic Mechanisms of Sensory Gating

#### Genetic Studies

In a family study (Freedman et al., 2006), the P50 sensory gating level was found to be genetically linked to 15q13–14.3, which contains the alpha 7 nicotinic receptor subunit (CHRNA7) gene.

In hominids, CHRNA7 gene was partially duplicated, forming a new gene, CHRFAM7A, the product of which acts as a negative regulator of 7alpha function. A 2-bp deletion in exon 6 of CHRFAM7A (rs67158670) enhances its inhibitory activity (Araud et al., 2011). It was shown that the presence of at least one 2-bp deletion is associated with a deficit of P50 gating in healthy individuals (Flomen et al., 2013). In patients with schizophrenia, the contribution of this polymorphism to the mechanisms of sensory gating was found to be significantly less pronounced than in the control sample (Raux et al., 2002).

Leonard et al. (2002) screened the promoter zone of CHRNA7 and found multiple polymorphic patterns that displayed significant associations with sensory gating in the control group but not in patients with schizophrenia. However, Houy et al. (2004) failed to replicate these results. Moreover, in contrast to previous results, one of the polymorphisms in the CHRNA7 promoter region (-194C) showed a positive association with P50 suppression, which was observed in the total sample of control subjects and patients.

In some studies, there was no association between different single-nucleotide polymorphisms in the CHRNA7 gene and P50 parameters or suppression rates (Cabranes et al., 2013; Bertelsen et al., 2015), so the problem needs further investigation.

#### Pharmacological Studies

Nicotine intake (smoking) was found to enhance sensory gating in healthy individuals with baseline low rates of P50 suppression (mainly, in carriers of Val/Val variant of COMT rs4680 polymorphism) and in patients with schizophrenia, but not in bipolar disorder (Whitton et al., 2021). In healthy persons, the effect of nicotine on P50 gating was relatively modest and was significantly enhanced after combined nicotine and MAO-A inhibitor compared to placebo and to the nicotine-alone condition (Smith et al., 2015).

In an attempt to determine the types of receptors mediating the effect of nicotine on sensory filtration, Hong et al. (2007) studied the effects of moderate doses (titration) of varenicline and found an increase in the level of P50 suppression in patients with schizophrenia. Since varenicline in low doses activates the alpha-4-beta-2 subunit containing cholinergic receptors, it can be assumed that the mechanisms of sensory filtration in schizophrenia are associated with the activity of this type of high-affinity receptors. At the same time, genetic studies show that the activity of low-affinity α7 receptors is substantial for P50 suppression mechanisms in healthy subjects.

The existence of different mechanisms of the deficit of P50 suppression that are specific in healthy and schizophrenic individuals is also supported by the fact that only patients with schizophrenia who do acute cigarette smoking increase the correlation between prefrontal executive cognitive functioning and P50 suppression (Rabin et al., 2009).

## Conclusion

Although the results of genetic and pharmacological studies are not numerous in the literature, they indicate the relative specificity in the patterns of dopaminergic and cholinergic activities associated with the level of information gating in healthy and schizophrenic individuals. Some data also point to the expediency of developing the concept of the optimal level of sensory and sensorimotor activity.

This can help to identify specific patterns of attention disorders and executive functions in numerous psychopathologies.

An important condition for the success of such studies is the use of unified protocols in cohorts of healthy individuals and those with mental disorders.

## Author Contributions

AP: formulation of the idea of the review, search and analysis of literature, writing, and design of articles.

## Conflict of Interest

The author declares that the research was conducted in the absence of any commercial or financial relationships that could be construed as a potential conflict of interest. The handling editor ZS declared a past co-authorship with the author.

## Publisher's Note

All claims expressed in this article are solely those of the authors and do not necessarily represent those of their affiliated organizations, or those of the publisher, the editors and the reviewers. Any product that may be evaluated in this article, or claim that may be made by its manufacturer, is not guaranteed or endorsed by the publisher.
